# Fabrication of nanowire network AAO and its application in SERS

**DOI:** 10.1186/1556-276X-8-495

**Published:** 2013-11-21

**Authors:** Qi Jiwei, Li Yudong, Yang Ming, Wu Qiang, Chen Zongqiang, Peng Jingyang, Liu Yue, Wang Wudeng, Yu Xuanyi, Sun Qian, Xu Jingjun

**Affiliations:** 1 The MOE Key Laboratory of Weak-Light Nonlinear Photonics, Tianjin Key Laboratory of Photonics and Technology, TEDA Applied Physics School and School of Physics, Nankai University, Tianjin 300457, China

**Keywords:** Surface-enhanced Raman scattering, AAO, Nanowire network

## Abstract

In this paper, nanowire network anodized aluminum oxide (AAO) was fabricated by just adding a simple film-eroding process after the production of porous AAO. After depositing 50 nm of Au onto the surface, nanowire network AAO can be used as ultrasensitive and high reproducibility surface-enhanced Raman scattering (SERS) substrate. The average Raman enhancement factor of the nanowire network AAO SERS substrate can reach 5.93 × 10^6^, which is about 14% larger than that of commercial Klarite® substrates. Simultaneously, the relative standard deviations in the SERS intensities are limited to approximately 7%. All of the results indicate that our large-area low-cost high-performance nanowire structure AAO SERS substrates have a great advantage in chemical/biological sensing applications.

## Background

Surface-enhanced Raman scattering (SERS) as a powerful and sensitive technique for the detection of chemical and biological agents received more attention since single-molecule detection with SERS was confirmed [1,2]. The enhancement of Raman signal was mainly attributed to the electromagnetic enhancement on the metal surface which was induced by the surface plasmon resonance (SPR). To obtain the huge Raman enhancement, noble metal nanogap structures, especially of sub-10-nm gap structures, have attracted considerable scholarly attention, which can support strong SERS due to the existence of enormous electromagnetic enhancement in the gap of metal nanostructure [3-16]. The enormous electromagnetic enhancement in the gap of metal nanostructure is caused by the strong coupling of the SPR, which is called ‘hot spot’. Apart from having a huge Raman enhancement, the high-performance SERS substrates should also be uniform and reproducible. Taking into account the commercial application, the high-performance SERS substrates should also be low cost and should achieve high output. Fabrication of high-performance SERS substrates has been the focus of attention [3-16]. Many low-cost methods and techniques have been proposed, like self-assembly [17,18], indentation lithography [6,19-24], corroding ultrathin layer [25], and femtosecond laser fabrication [26-29]. However, to date, for the existence of many limits for these low-cost techniques, the fabrication of large-area high-performance SERS substrate with sub-10-nm gap size is still critical for the practical applications of SERS.

Porous anodized aluminum oxide (AAO) was widely used in the SERS substrate fabrication for the existence of large-area high-ordered array of nanopores and the simple production process. Porous AAO can be used directly as SERS substrate after depositing Au or Ag on the surface [30] and can also be used as template to fabricate ordered array nanostructure SERS substrate [31-36]. Previous studies have shown that nanorod array and nanowire network, with dense nanojunctions and nanogaps, can support stronger SERS than porous structures [37-41]. The question, whether the nanorod array and nanowire network structure can be fabricated just by making a simple change to the production process of porous AAO, has not attracted the researcher's attention.

In this work, a simple film-eroding process was added after the production process of porous AAO to fabricate large-area low-cost nanowire network AAO which can be used as high-performance SERS substrate after depositing 50 nm of Au onto its surface. The Raman spectra of benzene thiol on the nanowire network AAO SERS substrates are measured and the average Raman enhancement factors (EFs) are calculated. Comparing with the porous AAO SERS substrates, the Raman peak intensities and the average EFs of nanowire network AAO SERS substrates have a significant enhancement. The average EF of our sensitive SERS substrate can reach 5.93 × 10^6^, about 35 times larger than that of porous AAO SERS substrate and about 14% larger than that of Klarite® substrates (Renishaw Diagnostics, Glasgow, UK), which indicates an enormous electromagnetic enhancement that exists in the nanowire network AAO SERS substrate. Repeated measurements and spatial mapping show an excellent reproducibility of the nanowire network AAO SERS substrate. The relative standard deviations in the SERS intensities are limited to only approximately 7%. Comparing with other fabrication methods of the high-performance SERS substrates, our method based on the mature production process of porous AAO is simpler, has lower cost, and is easier for commercial production. Therefore, we believe that our nanowire network AAO SERS substrates have great potential for applications.

## Methods

### Sample fabrication

**Figure 1:**
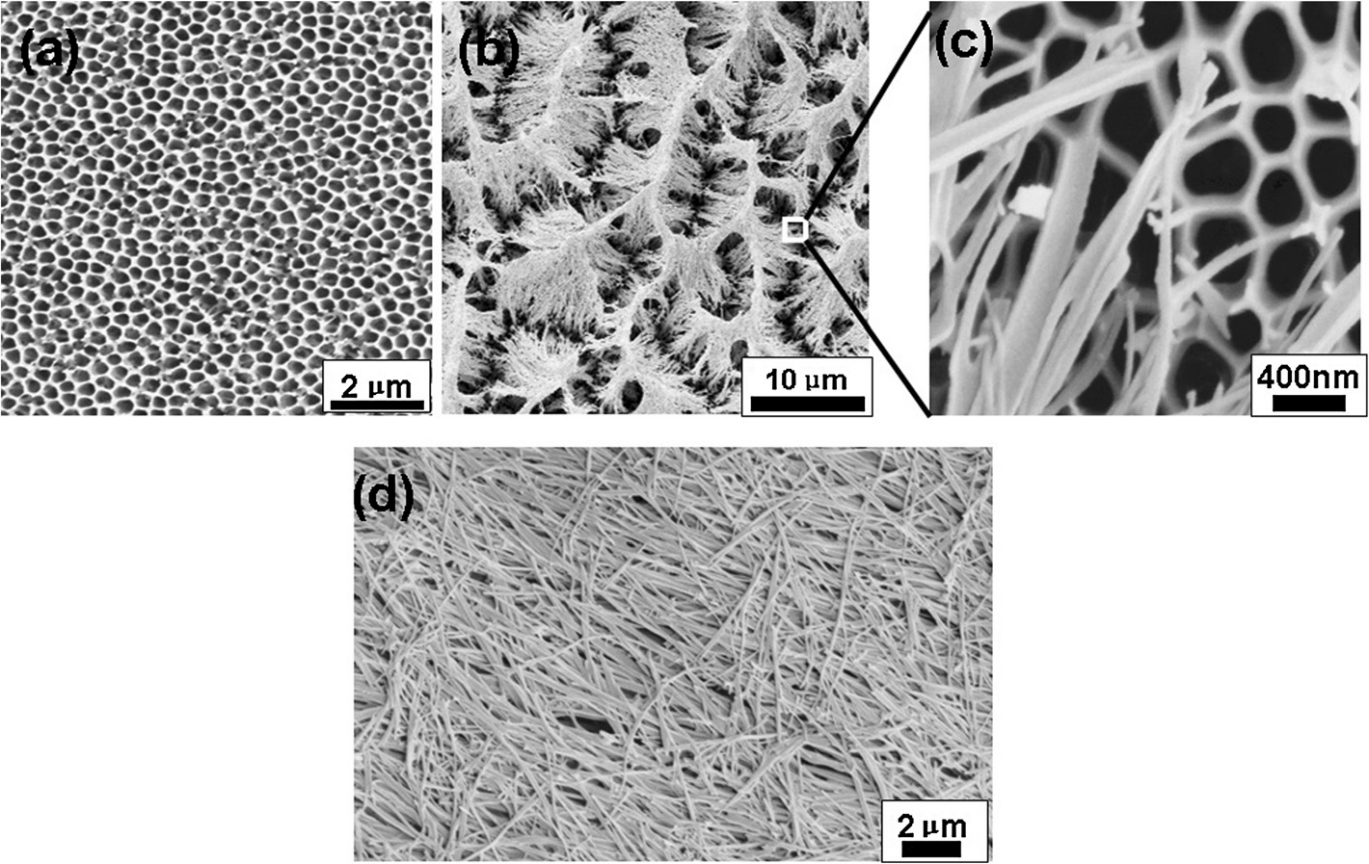
**SEM images of P-AAO (a), W-AAO1 (b), partial enlargement of W-AAO1 (c), and W-AAO2 (d).**

We commissioned Hefei Pu-Yuan Nano Technology Ltd to fabricate the porous AAOs and nanowire network AAOs. Production process [36] of porous AAO is already quite mature. The aluminum foil was first degreased with acetone under an ultrasonic bath for 10 min and then annealed at 350°C for 2 h. It was electropolished in a mixed solution (20% H_2_SO_4_ + 80% H_3_PO_3_ + 2% K_2_CrO_4_) under a constant voltage of 9 V and a temperature of 90°C to 100°C for 10 min. During this process, the aluminum was used as the anode and a platinum plate as the cathode. To obtain ordered nanopore arrays, we used a two-step anodizing process. The foil was anodized first in 0.3 M oxalic acid at 33 V at 0°C to 5°C for 14 h. It was then immersed in a mixed solution of 5.0 wt.% phosphoric acid and 1.8 wt.% chromic acid (1:1 in volume) at 60°C for 3 h to remove the alumina layer. In the second step, the sample was again anodized for 2 h under the same conditions and then, the underlying aluminum was removed in a CuCl_2_/HCl (13.5 g CuCl_2_ in 100 ml of 35% HCl) solution to expose the back-end AAO barrier. Finally, for pore widening, the sample was immersed in a 5.0 wt.% phosphoric acid solution at 30°C for 1 h. The scanning electron microscope (SEM) image of the fabricated porous AAO (sign with P-AAO) is present in Figure 1a. According the measurement result from the commercial software, the pore diameter and the pore spacing are approximately 302 ± 47 nm and 381 ± 52 nm, respectively.

To obtain the nanowire network AAOs, we required the manufacturer to add a film-eroding process after the pore-widening process. The P-AAOs were immersed again in mixed solution of 5.0 wt.% phosphoric acid and 1.8 wt.% chromic acid (1:1 in volume) at 60°C. The walls of the nanopores were damaged by the mixed acid solution, the nanopore structure fell down, and leaf-like nanowire cluster structure formed. Figure 1b shows the sample with a film-eroding time of 5 min, signed as W-AAO1. Figure 1c is the partial enlargement of W-AAO1, which show that the nanowire formed from the broken wall of nanopores. With further eroding, the nanowires formed from walls of nanopores became longer and thinner and could no longer prop each other. Therefore, the nanowire cluster fell down, and the nanowires lied on the surface as a uniform random layer. Figure 1d is the SEM image of the AAO with a film-eroding time of 10 min, called W-AAO2. The average diameter of nanowire on W-AAO1 and W-AAO2 was measured to be 68 ± 16 nm and 57 ± 15 nm, respectively. As shown in Figure 1b,d, dense junctions between the nanowires exist in W-AAO1 and W-AAO2. Previous studies have certificated that great amount of sub-10-nm gaps exist in these nanowire network structures [39-41].

After depositing 50 nm of Au onto the surface of P-AAO, W-AAO1, and W-AAO2, large-area high-performance SERS substrates were fabricated and were assigned as P-AAO-Au, W-AAO1-Au, and W-AAO2-Au, respectively.

### Detail of SERS spectra measurement

The measurement of SERS is same with our previous work [42]. Benzene thiol was used as the probe molecule. To ensure that a complete self-assembled monolayer (SAM) of benzene thiol was formed on the substrate surface, all of the SERS substrates were immersed in a 1 × 10^-3^ M solution of benzene thiol in ethanol for approximately 18 h and were subsequently rinsed with ethanol and dried in nitrogen [8,42]. All the Raman spectra were measured with a confocal Raman spectroscopic system (model inVia, Renishaw Hong Kong Ltd., Kowloon Bay, Hong Kong, China). The spectrograph uses 1,200 g mm^-1^ gratings, a 785-nm laser and a scan type of SynchroScan. The incident laser power was set to be 0.147 mW for all SERS substrates. All the SERS spectra were collected using × 50, NA = 0.5, long working distance objective and the laser spot size is about 2 μm. SERS spectra were recorded with an accumulation time of 10 s. After the SAM of benzene thiol was formed on the substrate surface, a single scan was performed. To get an accurate approximation of the enhancement factors, we measured the neat Raman spectrum of benzene thiol. For the measurement of the neat Raman spectrum of benzene thiol, the power of the 785-nm laser was 1.031 mW, the accumulation time was 10 s, the spot size was 20 μm, and the depth of focus was 18 μm.

**Figure 2:**
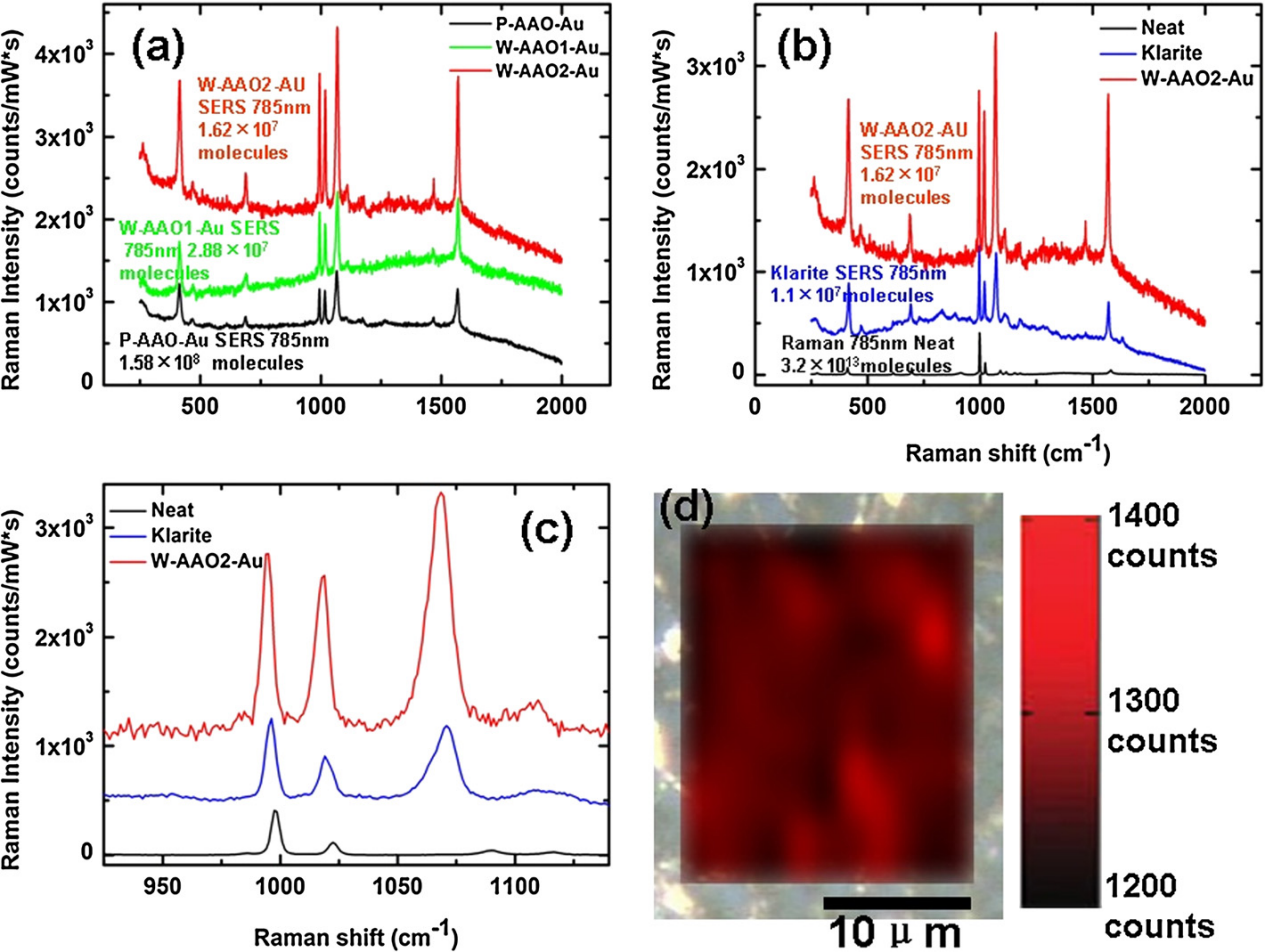
**Comparison of substrates and neat benzene thiol**, **Raman spectra, and spatial mapping. (a)** Comparison of the SERS of substrates P-AAO-Au, W-AAO1-Au, and W-AAO2-Au. **(b)** Comparison of the SERS of substrates W-AAO2-Au (red), Klarite® (blue), and neat Raman spectra (black) of benzene thiol collected at 785-nm incident. **(c)** Zoomed-in region of the spectra showing the three primary modes located near 1,000 cm^-1^, with the 998 cm^-1^ used for calculation of the SERS enhancement factor. The number of molecules of benzene thiol that each measurement is probing is denoted in the figure. **(d)** Spatial mapping of the SERS intensity at 998 cm^-1^ of SERS substrate W-AAO2-Au over an area larger than 20 μm × 20 μm. The background is the optical reflection image of substrate W-AAO2-Au photographed through a microscope with a × 50 objective.

Figure 2a shows the Raman spectra of the benzene thiol SAM on the P-AAO-Au (black), W-AAO1-Au (green), and W-AAO2-Au (red) with all having been normalized to account for the accumulation time and laser power. To characterize the SERS performance of our substrates, commercial Klarite® substrates were used as reference samples which consists of gold-coated textured silicon (regular arrays of inverted pyramids of 1.5-μm wide and 0.7-μm deep) mounted on a glass microscope slide. Figure 2b shows the normalized Raman spectra of the benzene thiol SAM on the W-AAO2-Au (red), on the Klarite® substrate (blue), and neat thiophenol (black).

### The calculation of EF

The average EFs were calculated from the following equation [8,42]:

$$\mathrm{EF}=\left(I_{\mathrm{SERS}}/I_{\mathrm{Raman}}\right)\times \left(N_{\mathrm{Raman}}/N_{\mathrm{SERS}}\right)$$

where *I*_SERS_ and *I*_Raman_ are the normalized Raman intensity of SERS spectra and neat Raman spectrum of benzene thiol, respectively. *N*_SERS_ and *N*_Raman_ represent the numbers of molecules contributing to SERS signals and neat Raman signals of benzene thiol, respectively. *I*_SERS_ and *I*_Raman_ can be measured directly from the Raman spectra. *N*_Raman_ is defined as follows [42]:

$$N_{\mathrm{Raman}}=\rho \times V\times \frac{N_{A}}{\mathrm{MW}}$$

where *ρ* = 1.073 g mL^-1^ and *MW* = 110.18 g mol^-1^ are the density and molecular weight of benzene thiol, respectively, and *V* is the collection volume of the liquid sample monitor. *N*_A_ is Avogadro's number. *N*_SERS_ is defined as follows [42]:

$$N_{\mathrm{SERS}}=\rho_{\mathrm{surf}}\times N_{A}\times S_{\mathrm{surf}}$$

where *ρ*_surf_ is the surface coverage of benzene thiol which has been reported as approximately 0.544 nmol cm^-2^[8,42], and *S*_surf_ is the surface area irradiated by exciting laser. For a clear comparison, *N*_SERS_ and *N*_Raman_ were quoted within Figure 2.

As shown in Figure 2, the average EFs based on the neat benzene thiol are dependent on the choice of Raman mode strongly. However, the relative Raman enhancement between our SERS substrates (including Klarite® substrate) was found to be relatively independent on the choice of Raman mode used for comparison. For comparison, the three Raman modes associated with vibrations about the aromatic ring are presented in Figure 2c. So, to get an accurate and comparable estimation of the average enhancement factor, Raman mode used for the calculation of the average EF must be selected carefully. Here, the intensities of the peak found at 998 cm^-1^, carbon-hydrogen wagging mode which is the furthest mode removed from the gold surface were used to compute the average EFs [8,42]. In addition, the average EF of Klarite® substrate was calculated to be 5.2 × 10^6^, which is reasonable because the enhancement factor for the inverted pyramid structure of Klarite® substrates relative to a non-enhancing surface is rated to a lower bound of approximately 10^6^[42].

## Results and discussion

The average peak intensity at 998 cm^-1^, the number of molecules contributing to the Raman signal, the calculated average EFs, and the relative standard deviation (RSD) for all SERS substrates are presented in Table 1. For each substrate, more than 80 spectra were collected at various positions to ensure that a reproducible SERS response was attained. Spatial mapping with an area larger than 20 μm × 20 μm of the SERS intensity of W-AAO2-Au was shown in Figure 2d as an example.

**Table 1:** SERS performance parameters of SERS substrates

**Sample**	**Peak intensity (counts/mW/s)**	**Number of molecules**	**Average EF**	**RSD (%)**
P-AAO-Au	351.62	1.58 × 10^8^	1.65 × 10^5^	8.02
W-AAO1-Au	997.92	2.88 × 10^7^	2.56 × 10^6^	8.25
W-AAO2-Au	1295.04	1.62 × 10^7^	5.93 × 10^6^	6.43
Klarite®	772.58	1.10 × 10^7^	5.21 × 10^6^	7.12

The average peak intensity at 998 cm^-1^, the calculated number of molecules, the average EFs and the RSD for P*-*AAO*-*Au, W*-*AAO1-Au, W*-*AAO2-Au, and Klarite® SERS substrates.

As shown in Figure 2a,b,c and Table 1, an obvious enhancement of Raman signal of the nanowire network AAO SERS substrates (W-AAO1-Au and W-AAO2-Au) is found, compared to that of porous AAO SERS substrate (P-AAO-Au). The Raman signal of W-AAO2-Au is the strongest in all of the SERS substrates (including the Klarite® substrate). Table 1 also shows a tremendous increase of average EF of the nanowire network AAO SERS substrate comparing with porous AAO SERS substrate. The average EFs of W-AAO1-Au and W-AAO2-Au are 2.56 × 10^6^ and 5.93 × 10^6^, about 14 and 35 times larger than that of P-AAO-Au (1.56 × 10^5^), respectively. Moreover, the average EF of our best SERS substrate, W-AAO2-Au, is larger than that of commercial Klarite® substrate by about 14%. The enormous average EFs of the nanowire network AAOs SERS substrates suggest that a gigantic electromagnetic enhancement occurs in the dense ‘hot junctions’ between the nanowires which exist in W-AAO1-Au and W-AAO2-Au. Additionally, the higher density of hot junctions that exist in W-AAO2-Au is the reason the peak intensity and the average EF of W-AAO2-Au are larger than that of W-AAO1-Au.

The spatial mapping with an area larger than 20 μm × 20 μm of the SERS intensity of W-AAO2-Au as shown in Figure 2d and the RSDs that are shown in Table 1 point out that the nanowire structure AAOs, especially W-AAO2-Au, are very uniform. Comparing with the RSD of P-AAO-Au, the RSD of W-AAO1-Au is larger, which is caused by the non-uniform leaf-like nanowire cluster structure on the surface of W-AAO1-Au, and the RSD of W-AAO2-Au is smallest, which can be attributed to the uniform random nanowire network structure formed on the surface of W-AAO2-Au. The reproducibility of our best SERS substrate, W-AAO2-Au, is even better than that of commercial Klarite® substrate. The RSDs of W-AAO2-Au in the SERS intensities were limited to only approximately 7% within a given substrate (that of Klarite® substrate is 7.12%), and the maximum deviation in the SERS intensities was limited to approximately 13%. The SERS response at a given point on the substrate was found to be highly reproducible, with variations in the detected response being limited to about 5%.

## Conclusions

In conclusion, we provide a simple, low-cost, and high output method, based on the riper production process of porous AAO, to fabricate large-area nanowire structure AAO which can be used as high-performance SERS substrate. The measured Raman spectra and the calculated average EFs show that compared with the porous AAO and commercial Klarite® substrates, the nanowire structure AAO SERS substrates are sensitive and uniform in large area. The average EF of our sensitive SERS substrate can reach 5.93 × 10^6^, which indicates the existence of enormous electromagnetic enhancement in the nanowire network AAO substrate. Repeated measurements and spatial mapping show an excellent uniformity of the nanowire network AAO substrate. The RSDs in the SERS intensities of W-AAO2-Au are limited to approximately 7%. For these superiorities, we believe that our nanowire structure AAO SERS substrates are suitable choice for chemical/biological sensing applications.

## Abbreviations

AAO: Anodized aluminum oxide; EF: Raman enhancement factor; RSD: Relative standard deviation; SAM: Self-assembled monolayer; SEM: Scanning electron microscope; SERS: Surface-enhanced Raman scattering; SPR: Surface plasmon resonance.

## Competing interests

The authors declare that they have no competing interests.

## Authors' contributions

QJ conceived of the study, carried out the fabrication of the SERS substrates, the measurement and analysis, the simulation, and drafted the manuscript. LY (Yudong) participated in the SERS spectra analysis and discussion. YM, PJ, LY (Yue), and WQ participated in the SEM measurements and SERS spectra measurements. CZ, WW, and YX participated in the fabrication of the SERS substrates. XJ and SQ were the PI of the project and participated in the design and coordination of the study and revised the manuscript. All authors read and approved the final manuscript.

## Authors' information

QJ is a lecturer at Nankai University. His research interest includes fabrication of the nanostructure, nonlinear optical properties of nanostructures, fanoresonance, and surface plasmon resonance and their applications in SERS, sensor, and so on.
